# Glucagon-like peptide-1 receptor agonists and rotator cuff disease: a scoping review

**DOI:** 10.1186/s12891-026-10092-9

**Published:** 2026-06-15

**Authors:** Dave Osinachukwu Duru, Andrew Kailin Zhou, David Kwan Ryung Lee, Patrick J. Carroll, Amr Elmaraghy

**Affiliations:** 1 School of Clinical Medicine, University of Cambridge, Cambridge, UK; 2 University of Warwick, Coventry, UK; 3 Department of Trauma and Orthopaedics, University Hospital Coventry and Warwickshire, Coventry, UK; 4 Division of Orthopaedic Surgery, Department of Surgery, University of Toronto, Toronto, ON Canada; 5 Surgeon Investigator, Li Ka Shing Knowledge Institute, Unity Health Toronto, Toronto, ON Canada

**Keywords:** Glucagon-like peptide-1 receptor agonist, GLP-1, Rotator cuff, Rotator cuff repair, Obesity, Diabetes, Tendon healing

## Abstract

**Background:**

Obesity and type 2 diabetes mellitus (T2DM) are associated with impaired rotator cuff tendon healing and inferior clinical outcomes following surgical repair. Glucagon-like peptide-1 receptor agonists (GLP-1RA) are increasingly used to manage obesity and T2DM. However, their influence on rotator cuff disease biology and outcomes following cuff repair remains unknown.

**Methods:**

A scoping review was conducted in accordance with PRISMA-ScR guidelines. MEDLINE, Embase, PubMed, and the Cochrane Library were searched from inception to 28 January 2026 for studies evaluating associations between GLP-1RA exposure and rotator cuff disease. Eligible studies included human observational cohort studies and preclinical experimental studies. Data were extracted independently by two reviewers and synthesised descriptively.

**Results:**

Six studies were included, four human retrospective cohort studies and two controlled preclinical studies. The human studies included 1,481,529 patients, of which 391,716 were GLP-1RA users. Preoperative GLP-1RA use was not associated with increased postoperative complications following rotator cuff repair. However, GLP-1RA use was associated with increased incidence of atraumatic rotator cuff tears and subsequent repair. Preclinical models of acute rotator cuff injury and repair found that liraglutide improved biomechanical strength and organisation of the repaired tendon–bone interface, reduced fatty infiltration, and attenuated cellular stress responses.

**Conclusions:**

The current evidence does not demonstrate an association between GLP-1RA use and increased risk of postoperative complications following rotator cuff repair. Studies reported an association between GLP-1RA use and higher incidence of atraumatic rotator cuff tears. Preclinical data suggest GLP-1RA may influence rotator cuff tissue healing. High-quality prospective studies are required to clarify the effects of GLP-1RA on rotator cuff disease.

**Supplementary Information:**

The online version contains supplementary material available at 10.1186/s12891-026-10092-9.

## Background

Rotator cuff tears are a leading cause of shoulder pain and functional disability in individuals over the age of 50 years [1]. Management may be non-operative or surgical, with both open and arthroscopic rotator cuff repair leading to substantial improvements in pain and functional outcomes [2–6]. Despite advances in surgical technique and rehabilitation, postoperative outcomes remain variable, influenced by patient-related factors like socioeconomic status, tear chronicity, and metabolic health [6–9]. Type 2 diabetes mellitus (T2DM) and obesity are both associated with poor rotator cuff repair outcomes, including higher postoperative complication rates and increased risk of re-tear [10, 11]. This underscores the importance of metabolic optimisation including glycaemic control and weight management in patients with rotator cuff disease.

Glucagon-like peptide-1 receptor agonists (GLP-1RA) have emerged as therapies for the management of T2DM and obesity [12]. Due to their expanding use, orthopaedic surgeons are increasingly likely to encounter patients receiving these agents before or after rotator cuff repair [13]. Beyond their glucose-lowering and weight-reducing effects, GLP-1RA exert pleiotropic actions, including suppression of inflammatory pathways, antioxidant effects, and reductions in systemic metabolic stress [14]. GLP-1 receptors (GLP-1Rs) have been identified in musculoskeletal tissues, such as the rotator cuff, where receptor activation modulates extracellular matrix remodelling, reduces oxidative stress, attenuates pro-inflammatory cytokine signalling, and promotes collagen organisation at the tendon–bone interface [15–17]. These mechanisms suggest a biologically plausible basis for GLP-1RA as modifiers of rotator cuff tendon, muscle, and tendon–bone healing biology. However, whether these experimental observations translate into clinically meaningful differences in rotator cuff disease incidence, postoperative complications, structural healing, or functional recovery in humans remains uncertain.

The relationship between GLP-1RA exposure and rotator cuff disease is therefore clinically relevant but incompletely defined. Existing evidence spans heterogeneous human database studies and preclinical models, addressing different outcomes including incident rotator cuff tears, complications after repair, tendon–bone healing, fatty infiltration, and molecular stress pathways. To date, this emerging literature has not been systematically mapped. Accordingly, this scoping review aimed to summarise the available clinical and preclinical evidence evaluating associations between GLP-1RA exposure and rotator cuff disease, including incident disease, postoperative outcomes following rotator cuff repair, and experimental models of rotator cuff injury and healing.

## Methods

This scoping review was conducted to assess the influence of GLP-1RA on rotator cuff disease. The methodology adhered to the Preferred Reporting Items for Systematic Reviews and Meta-Analyses Extension for Scoping Reviews (PRISMA-ScR) guidelines, which provide a structured framework for synthesising heterogeneous and emerging bodies of literature and identifying gaps in evidence [18]. The review protocol was developed a priori and followed throughout the study, with no subsequent changes.

### Search strategy

A comprehensive search was conducted of MEDLINE (via Ovid), Embase (via Ovid), PubMed, and the Cochrane Library from their inception to January 28, 2026. To identify relevant literature, keywords included terms related to GLP-1RA (“glucagon-like peptide-1 receptor agonist”, “GLP-1”, “GLP-1RA”, “liraglutide”, “exenatide”, “semaglutide”, “dulaglutide”) and rotator cuff disease (“rotator cuff”, “rotator cuff tear”, “rotator cuff disease”, “shoulder pathology”, “supraspinatus”). The search strategy was developed with a medical librarian and the search for each of the databases is provided in Supplementary Table 1. Reference lists of retrieved articles and prior reviews were also screened for additional sources.

All search results were imported into Rayyan (Cambridge, MA, USA) and duplicates were removed. Two reviewers independently screened titles and abstracts against the inclusion criteria, blinded to the decisions of the other reviewer. Two reviewers independently examined full-text articles to determine final inclusion. Any disagreements at title and abstract or full-text screening were resolved through discussion or consultation with a third reviewer.

### Eligibility criteria

Included studies evaluated GLP-1RA exposure in the context of rotator cuff disease and/or rotator cuff repair biology. Given the early and heterogeneous nature of the literature, human and preclinical studies were included as separate evidence streams. The human evidence stream was intended to map clinical associations between GLP-1RA exposure and rotator cuff-related outcomes, including: (1) the incidence of rotator cuff tears, and (2) postoperative outcomes following rotator cuff repair among GLP-1RA users compared with non-users. The preclinical evidence stream was intended to map experimental biological mechanisms by which GLP-1RA may influence rotator cuff tendon, muscle, or tendon–bone interface biology in controlled animal models. Accordingly, eligible human studies included observational cohort, case-control, pharmacovigilance analyses, database, and other comparative clinical studies evaluating GLP-1RA exposure in patients with rotator cuff disease or patients undergoing rotator cuff repair. Eligible preclinical studies included controlled in vivo animal experiments evaluating GLP-1RA exposure in rotator cuff injury, degeneration, or repair models. In vitro studies were eligible only when they directly examined rotator cuff relevant mechanisms and were linked to an included in vivo experimental model. Human and preclinical findings were extracted and synthesised separately, with the human studies used to evaluate clinical associations and postoperative outcomes, and the preclinical studies used to contextualise potential biological mechanisms. No age restrictions were imposed for human studies. Excluded studies included conference abstracts without extractable data, review articles, editorials, commentaries, non-English studies without an English translation, and studies that evaluated GLP-1RA exposure without any rotator cuff-relevant outcome or mechanism.

### Data extraction and synthesis

Data extraction was independently conducted by two reviewers using a predefined data collection form in Microsoft Excel (version 16.100). Key extracted data for human studies included study design, population characteristics (e.g. age, sex), sample size, comorbidities, GLP-1RA exposure window, matching variables, type of rotator cuff repair, postoperative outcomes, re-tear rates, follow-up, and postoperative complications. Risk ratios (RRs), odds ratios (ORs), or hazard ratios (HRs), with 95% confidence intervals (CIs), were extracted when available. Significance was defined as *p* < 0.05. Key extracted data from preclinical studies included model type (e.g. rat, cell), method of induced rotator cuff injury, signalling pathways investigated and identified, histological findings, and biomechanical findings. Any discrepancies in data extraction were resolved through discussion or consultation with a third reviewer. The extracted data were synthesised descriptively.

### Risk of bias

Two reviewers independently assessed the risk of bias via Risk Of Bias In Non-randomized Studies of Interventions (ROBINS-I) for non-randomised human studies. Any discrepancies were resolved by discussion or consultation with a third reviewer. Two reviewers independently assessed the preclinical experimental studies using the Joanna Briggs Institute checklist for quasi-experimental studies. Discrepancies were resolved by discussion or consultation with a third reviewer.

## Results

**Fig. 1:**
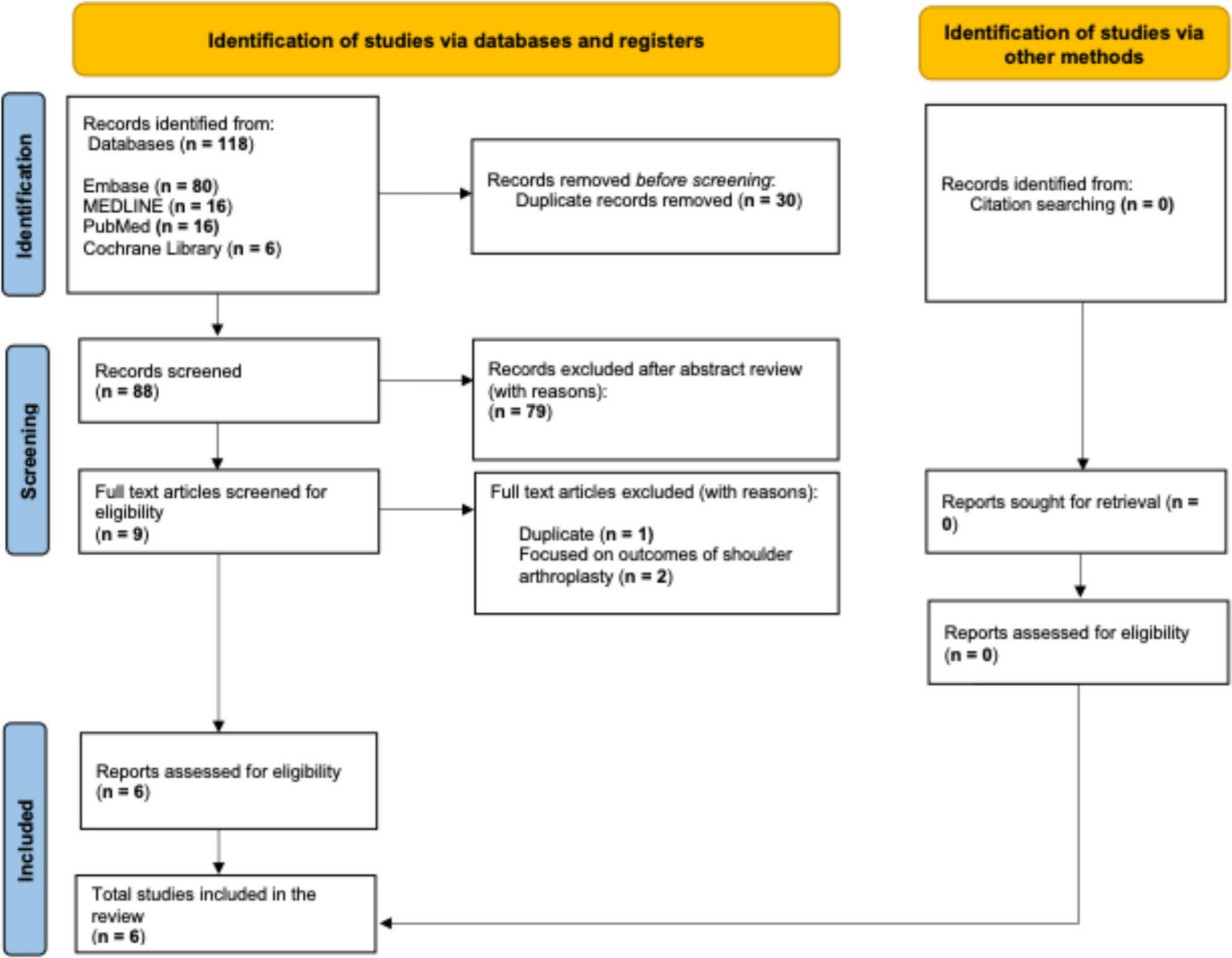
PRISMA Flow Diagram of Studies

A total of 118 records were identified through database searching. After removal of duplicates, 88 studies underwent title and abstract screening, of which 79 were excluded. Nine studies were assessed in full text, and six met the inclusion criteria for final analysis [17, 19–23] (Fig. 1). Included studies were published between 2024 and 2026.

### Characteristics of included human studies

Four studies were retrospective cohort studies, collectively including 1,481,529 patients, of whom 391,716 were GLP-1RA users matched to non-users (Table 1). Across all studies, matching was performed using propensity-based methods that incorporated key demographic and clinical variables, including age, sex, BMI, HbA1c, tobacco use, and medications (e.g. insulin and metformin). Two studies [20, 23] assessed postoperative complications, reoperation, or re-tear following rotator cuff repair among GLP-1RA users compared with non-users, while two studies [19, 22] evaluated associations between GLP-1RA exposure and incident rotator cuff tears or subsequent repair. Su et al. (2024) [22] employed an active-comparator design using sodium–glucose cotransporter-2 inhibitors (SGLT-2i).

**Table 1:** Characteristics of Included Human Studies

Authors and Year	Study design	Matched sample size (% Female)	Mean age, years (SD)	Follow-up	GLP-1RA(s) used	Outcomes measured
Rasmussen & Ilyas, 2025 [23]	RC	GLP-1RA user: 5306 (51%) GLP-1RA nonuser: 5306 (51%)	59 ± 9 (both groups)	12 months	Semaglutide, dulaglutide, liraglutide	Reoperation risk at 3, 6, and 12 months; readmission; septic arthritis; adhesive capsulitis; PE; DVT; shoulder bursitis
Seddio et al., 2025 [20]	RC	GLP-1RA user: 1094 (51%) Non-user: 4110 (51%)	GLP-1RA user: 57.6 ± 7.2 Non-user: 57.6 ± 7.1	Minimum 2 years	Semaglutide	90-day adverse events, severe adverse events, minor adverse events, ED visits, readmission, revision surgery, and 2-year re-tear
Su et al., 2024 [22]	RC	GLP-1RA user: 274,026 (47%) SGLT2i user: 274,026 (47%)	59.5 (both groups)	Mean 1.8 years (SGLT2i) and 2.0 years (GLP-1RA)	Semaglutide	Risk of rotator cuff tear and risk of receiving rotator cuff repair surgery
Davis et al., 2026 [19]	RC	Nonobese DM: 37,438 GLP-1RA users vs. 37,438 non-users; female 53% in both groups Obese DM: 63,164 GLP-1RA users vs. 63,164 non-users; female 54% in both groups Obese No DM: 10,688 GLP-1RA users vs. 10,688 non-users; female 70% in both groups.	Nonobese DM: GLP-1RA: 57.7 ± 11.7; Non-user: 57.5 ± 12.2 Obese DM: GLP-1RA user: 56.6 ± 11.6; Non-user: 56.3 ± 12.7 Obese No DM: GLP-1RA: 50.4 ± 12.9; Non-user: 50.5 ± 13.9	Minimum 5 years	Semaglutide, albiglutide, dulaglutide, liraglutide, lixisenatide, exenatide	Incidence of atraumatic rotator cuff tears

*RC *Retrospective matched cohort study, *Obese No DM *obese non-diabetic cohort, *Obese DM *obese diabetic cohort, *Nonobese DM *non-obese diabetic cohort, *GLP-1RA *Glucagon-like peptide 1 receptor agonist, *SGLT2i *sodium-glucose transport 2 inhibitor, *ED *emergency department, *RCR *rotator cuff repair, *DVT *deep vein thrombosis, *PE *pulmonary embolism

Across cohorts, approximately 49% of patients were female, and mean cohort ages ranged from 50.4 to 59.5 years. The majority of patients had T2DM. Reported GLP-1RA included semaglutide, dulaglutide, liraglutide, albiglutide, lixisenatide, and exenatide.

### Definition of GLP-1RA exposure

Definitions of GLP-1RA exposure varied significantly across the four cohort studies, with the findings presented in Supplementary Table 2. In studies evaluating outcomes following rotator cuff repair, exposure was defined relative to the timing of surgery. One study required at least three months of continuous preoperative GLP-1RA use to ensure adequate exposure [23], while another defined exposure as documented semaglutide use within the year preceding arthroscopic repair [20]. In contrast, studies examining the incidence of rotator cuff disease employed longitudinal exposure definitions anchored to medication initiation [19, 22].

### Clinical postoperative outcomes following rotator cuff repair

Across the included human cohort studies, preoperative GLP-1RA exposure was not associated with an increased risk of postoperative complications or reoperation following rotator cuff repair. Rasmussen & Ilyas (2025) [23] reported no significant difference in reoperation risk between GLP-1RA users and non-users at 3 months (RR 0.78, 95% CI 0.55 to 1.09, *p* = 0.1409), 6 months (RR 0.97, 95% CI 0.76 to 1.24, *p* = 0.7987), and 12 months (RR 0.98, 95% CI 0.82 to 1.17, *p* = 0.8500). No significant differences were identified for septic arthritis, adhesive capsulitis, pulmonary embolism, deep vein thrombosis, shoulder bursitis, or readmission. In contrast, Seddio et al. (2025) [20] reported lower postoperative adverse event rates among semaglutide users with T2DM undergoing arthroscopic rotator cuff repair. Any 90-day adverse event occurred in 11.0% of semaglutide users compared with 27.4% of non-users; multivariable analysis showed higher odds of any adverse event among non-users (OR 3.65, 95% CI 2.96 to 4.53, *p* < 0.001). Emergency department visits were also higher among non-users (41.4% vs. 24.2%, OR 2.51, 95% CI 2.14 to 2.95, *p* < 0.001). Two-year re-tear rates were lower in semaglutide users compared to non-users (12.5% vs. 18.3%, *p* < 0.001).

### Incidence of rotator cuff disease

Two human cohort studies evaluated the association between GLP-1RA exposure and incident rotator cuff disease. Davis et al. (2026) [19] reported a higher 5-year incidence of atraumatic rotator cuff tears among GLP-1RA users across all three matched metabolic subgroups: the nonobese diabetic cohort (3.7% vs. 2.3%, HR of 1.582, 95% CI 1.452 to 1.724), obese diabetic cohort (4.5% vs. 3.3%, HR of 1.357, 95% CI 1.281 to 1.438), and obese nondiabetic cohort (3.2% vs. 2.3%, HR of 1.379, 95% CI 1.170 to 1.627). Su et al. (2024) [22] reported similar directionality comparing SGLT2 inhibitors with GLP-1RA. Compared with GLP-1RA use, SGLT2 inhibitor use was associated with a lower risk of rotator cuff tear (HR 0.812, 95% CI 0.761 to 0.867) and rotator cuff repair surgery (HR 0.900, 95% CI 0.815 to 0.994). As GLP-1RA were the reference group, these findings may imply a relatively higher associative risk of both rotator cuff tear and subsequent repair among GLP-1RA users.

### Characteristics of included rat studies

Two controlled laboratory studies evaluated liraglutide in rat models of rotator cuff injury and repair (Table 2). Both studies used surgically induced supraspinatus tendon injury followed by immediate repair, representing acute traumatic repair models. Yoon et al. (2025) [21] used 40 male 12-week-old Sprague Dawley rats and randomly assigned animals to control or liraglutide treatment groups, with tissue harvest at 6 or 12 weeks. Liraglutide was administered subcutaneously from postoperative day 1 and continued daily until the planned endpoint. Zhang et al. (2026) [17] used 24 male 10-week-old Sprague Dawley rats randomised to sham, tendon transection with saline, or tendon transection with liraglutide groups, with tissue harvest at 4 weeks after repair. Reporting of internal validity safeguards varied between studies. While both studies reported random allocation to treatment groups, neither clearly described allocation concealment, attrition, exclusions, or handling of missing data. Yoon et al. (2025) [21] reported blinded assessment for functional outcomes, including range of motion and wire hanging tests, and included an a priori sample size calculation. Zhang et al. (2026) [17] reported blinded histological scoring by three investigators using the modified Bonar scale but did not report a sample size calculation for the animal experiment, and blinding was not clearly described for biomechanical, molecular, or immunohistochemical outcomes.

**Table 2:** Characteristics of Included Rat Studies

Authors and Year	Animal model and disease relevance	Liraglutide timing and duration	Groups and harvest timing	Outcomes measured
Yoon et al., 2025 [21]	40 male Sprague Dawley rats, 12 weeks old. Supraspinatus tendon transection and immediate repair.	Liraglutide 250 µg/kg/day subcutaneously. Injections started on postoperative day 1 and continued until sacrifice.	Four subgroups, 10 rats each: control 6 week, control 12 week, liraglutide 6 week, liraglutide 12 week. Tissue harvest at 6 and 12 weeks.	Fatty infiltration, wet muscle weight, qRT PCR gene expression, range of motion, wire hanging test, electromyography.
Zhang et al., 2026 [17]	24 male Sprague Dawley rats, 10 weeks old. Supraspinatus tendon transection and immediate repair.	Liraglutide 200 µg/kg/day subcutaneously after surgery and continued daily until sacrifice.	Three groups, 8 rats each: sham skin incision with saline, operation with tendon transection and saline, and operation with tendon transection and liraglutide. Tissue harvest at 4 weeks.	Tendon cell inflammation, apoptosis, endoplasmic reticulum stress, histology, modified Bonar score, collagen organisation, immunohistochemistry, and biomechanical testing.

*ROM* range of motion, *ER *endoplasmic reticulum, *qRT-PCR *Quantitative Reverse Transcription Polymerase Chain Reaction

### Biomechanical and functional outcomes

Zhang et al. (2026) [17] performed load-to-failure testing on supraspinatus tendon–humerus complexes after euthanasia, with tendons secured in sandpaper grips and tested at 1 mm/s under continuous saline irrigation. They reported higher failure load and stiffness in the liraglutide-treated group compared with the saline-treated group.

Yoon et al. (2025) [21] evaluated postoperative shoulder function using range of motion testing, wire hanging performance, and electromyography. They reported improved internal rotation at 6 weeks in the liraglutide group compared with controls, *p* < 0.001, while external rotation did not differ at 6 weeks, *p* = 0.939. At 12 weeks, external rotation was improved in the liraglutide group. Muscle strength assessed using the wire hanging test was also reported to improve in the liraglutide group. In contrast, compound muscle action potential amplitude was higher in the liraglutide group at both 6 weeks (8.92 ± 3.97 ms·mV vs. 2.20 ± 1.88 ms·mV, *p* < 0.001) and 12 weeks (9.66 ± 5.65 ms·mV vs. 1.98 ± 1.83 ms·mV, *p* < 0.01).

### Histologic and structural healing outcomes

Zhang et al. (2026) [17] evaluated tendon–bone interface histology using H&E, Masson’s trichrome, and Sirius red staining of supraspinatus tendon–humeral complexes. They reported that liraglutide-treated animals demonstrated improved collagen organisation, reduced inflammatory infiltration, and more preserved tendon–bone interface microarchitecture compared with saline-treated operated controls.

Yoon et al. (2025) [21] evaluated supraspinatus fatty infiltration using Oil Red O staining and quantitative image analysis. Fatty infiltration was reported to increase progressively after repair in control animals, whereas liraglutide-treated animals demonstrated significantly reduced fatty infiltration at both 6 and 12 weeks, *p* < 0.01. The authors reported that fatty infiltration was reduced by more than 10-fold at 12 weeks in the liraglutide group compared with controls, without a significant difference in muscle fibre cross-sectional area.

### Molecular pathways and cellular stress responses

Both preclinical studies reported molecular changes that may help explain the observed tissue-level findings, but these should be interpreted as pathway associations rather than definitive causal mediation. In Yoon et al. (2025) [21], liraglutide treatment was associated with increased mRNA expression of components of the PKA–CREB1–BDNF axis in supraspinatus muscle, including PKA at 6 and 12 weeks, *p* < 0.001 for both; CREB1 at 6 weeks, *p* < 0.001, and 12 weeks, *p* = 0.037; PGC-1α at 6 weeks, *p* = 0.026, and 12 weeks, *p* < 0.001; and BDNF at 6 weeks, *p* = 0.007, and 12 weeks, *p* = 0.046. In contrast, there were no significant differences in mRNA expression of atrophy-related genes, including Atrogin-1 and MuRF-1, adipogenic markers, such as PPAR-γ and C/EBP-α, or UCP1, a fat browning marker. These findings suggest that liraglutide-associated improvements in fatty infiltration and function may be linked to neurotrophic signalling, but the study did not establish causal mediation through PKA–CREB1–BDNF signalling.

Zhang et al. (2026) [17] reported that liraglutide was associated with increased GLP-1R expression, increased collagen I expression, reduced MMP13 expression, and reduced expression of cellular stress and apoptosis markers including BAX and CHOP in the repaired rotator cuff model. In vitro experiments further showed that liraglutide altered AMPK/SIRT1 pathway markers in tendon cells exposed to inflammatory or endoplasmic reticulum stress stimuli, and that pharmacological inhibition of AMPK or SIRT1 attenuated some of liraglutide’s cytoprotective effects. These experiments support involvement of the GLP-1R–AMPK/SIRT1 axis in tendon cell stress responses. However, because most pathway analyses were based on marker expression, in vitro perturbation, and association with treatment response, they should not be interpreted as definitive proof that this pathway causally leads to improved tendon–bone healing.

### Risk of bias

All human observational studies were critically appraised using ROBINS-I (Supplementary Table 3). Overall risk of bias was judged as moderate or serious. The main concerns related to confounding, as GLP-1RA users may differ from comparators in metabolic disease severity, healthcare engagement, prescribing indication, activity level, and likelihood of diagnostic imaging. Although studies used propensity matching, multivariable adjustment, or active-comparator designs, none captured key rotator cuff-specific prognostic variables, including tear size, chronicity, tendon quality, fatty infiltration, symptom severity, repair technique, or rehabilitation exposure. Concerns were also identified in classification of interventions because GLP-1RA exposure was based on prescription or medication codes, with limited information on dose, duration, adherence, treatment interruption, or perioperative discontinuation. Bias due to selection of participants, deviations from intended interventions, missing data, outcome measurement, and selection of reported results was generally judged as low to moderate. Outcome measurement was limited by reliance on administrative diagnosis and procedure codes.

The two preclinical experimental studies were appraised using the JBI quasi-experimental checklist (Supplementary Table 4). Both studies clearly defined the exposure and outcome, included control groups, used broadly appropriate statistical analyses, and measured outcomes similarly across groups. However, both were limited by short-term acute repair models in young male rats, which restricts generalisability to chronic degenerative rotator cuff disease in older humans. Additional concerns included incomplete reporting of allocation concealment, blinding across all outcome domains, attrition, and sample size justification. Accordingly, the experimental studies were considered methodologically acceptable for hypothesis-generating mechanistic evidence, but insufficient to support direct clinical inference.

## Discussion

This scoping review presents the clinical and preclinical evidence evaluating GLP-1RA use in the context of rotator cuff disease. The available literature remains limited but emerging, with all studies published between 2024 and 2026. Based on the current evidence, GLP-1RA use does not appear to be associated with increased postoperative complications following rotator cuff repair. However, the evidence suggests GLP-1RA use is associated with potentially increased incidence of atraumatic rotator cuff tears and need for subsequent repair. Limited preclinical evidence suggests that GLP-1RA may have a potential biological effect that supports rotator cuff healing following injury and repair.

Across large, propensity matched cohort studies, preoperative GLP-1RA use was not associated with increased postoperative complications following rotator cuff repair, with either neutral or positive associations reported [20, 23]. Such findings mirror the current arthroplasty landscape, which heterogeneously reports improved or neutral effects of GLP-1RA use on postoperative complication risks [24–30]. However, the two incidence-based studies found increased diagnosis of atraumatic rotator cuff tears and increased rates of cuff repair among GLP-1RA users compared to non-users [19], and specifically SGLT2i users [22]. The active-comparator findings involving SGLT2i require particularly cautious interpretation. Although active comparators may reduce some confounding compared with untreated control groups, SGLT2i and GLP-1RA users may still differ systematically in renal function, cardiovascular risk, obesity severity, diabetes phenotype, prescribing indication, medication tolerance, and clinician selection.

Nevertheless, the apparent association between GLP-1RA use and increased risk of atraumatic rotator cuff tears is not yet clearly explained in the literature. Potentially weight loss and improved metabolic health following GLP-1RA initiation may enable increased physical activity and shoulder loading [19]; coupled with increased lean muscle mass loss, this may increase the risk of rotator cuff tears. However, this is speculative and not supported by directly measured activity data. Additionally, surveillance bias may contribute to the observed association between GLP-1RA exposure and rotator cuff disease [19]. Patients prescribed GLP-1RA may have more frequent medical visits, medication monitoring, weight-management follow-up, and opportunities for diagnostic imaging than matched non-users, thereby increasing the likelihood that symptomatic or previously unrecognised rotator cuff pathology is detected and coded. Therefore, the higher observed incidence of rotator cuff tears among GLP-1RA users may partly reflect differential ascertainment rather than a true increase in tendon disease.

In rat models of supraspinatus injury, followed by repair, liraglutide use was associated with improved tendon–bone healing, enhanced biomechanical strength, decreased fatty infiltration, and preserved muscle function [17, 21]. The studies suggest several potential mechanisms for GLP-1RA to influence rotator cuff biology, including attenuation of cellular stress responses, modulation of inflammatory signalling, reduced apoptosis, improved extracellular matrix organisation, and preservation of muscle quality. However, these mechanisms map onto different components of rotator cuff pathology. Tendon–bone healing, fatty infiltration, muscle degeneration, and neuromuscular recovery are not interchangeable outcomes, and each may respond differently to metabolic or pharmacological modulation. Thus, while these findings support biological plausibility for GLP-1RA influencing aspects of rotator cuff repair biology, they should not be interpreted as evidence of a uniform beneficial effect on all determinants of recovery.

There are several limitations of this review. All human studies relied on administrative databases, precluding assessment of key surgical variables. For instance, no information is known pertaining to tear size, chronicity, tendon quality, fatty infiltration, symptom severity, imaging indication, repair technique, rehabilitation, or structural healing, all known to influence outcomes of rotator cuff repair. Coding also conflates the type of tear, whether incident, pre-existing, traumatic, atraumatic, or postoperative re-tears, all with differing indications and prognostic implications [31–35]. A further limitation is the potential for exposure misclassification. GLP-1RA exposure was defined using medication records or database codes, which may not reflect adherence, dose escalation, treatment interruptions, or perioperative discontinuation. Therefore, patients classified as exposed may have had variable exposure, while some patients classified as non-users may have later initiated therapy or received GLP-1RA outside the captured database window. This limits inference regarding dose, duration, timing relative to surgery, and whether any observed association is attributable to GLP-1RA itself. Immortal time bias is also important when interpreting incidence-based studies. Patients classified as GLP-1RA users must survive, remain observable, and have sufficient healthcare contact to receive a GLP-1RA prescription before being classified as exposed. If this exposure period is not appropriately accounted for, pre-exposure person-time may be misclassified as GLP-1RA-exposed follow-up, thereby biasing observed associations between GLP-1RA use and subsequent rotator cuff disease diagnosis or repair. Residual confounding by indication is unavoidable and should not be considered eliminated by propensity matching. GLP-1RA users differ systematically from non-users in obesity severity, diabetes duration, glycaemic control, medication history, healthcare access, and likelihood of undergoing clinical assessment or imaging. The use of administrative databases also limits conclusions regarding patient-reported and functional outcomes, including pain, strength, range of motion, return to activity, and satisfaction. Additionally, other important factors such as aspiration or sarcopenia risk with GLP-1RA use were not assessed by the included studies. Moreover, the preclinical evidence is derived from acute injury models in young healthy rats; this may not recapitulate the chronic degenerative biology that often characterises human rotator cuff disease. Furthermore, both preclinical studies only evaluated liraglutide and findings in the human studies were largely not stratified by drug type. This limits generalisability of findings to specific GLP-1RA, each with distinct pharmacological properties. Whether the observed associations reflect class-wide phenomena, agent-specific mechanisms, residual bias, or differences in patient selection therefore remains uncertain.

This review highlights several important knowledge gaps that should guide future research. Prospective clinical studies are needed to determine whether GLP-1RA exposure is associated with rotator cuff disease incidence, postoperative complications, structural healing, or functional recovery, while accounting for confounding by metabolic disease severity, healthcare utilisation, and surveillance intensity. Such studies should incorporate granular medication data, including agent type, dose, duration, adherence, timing of initiation, and postoperative continuation. Future studies should also include imaging-confirmed diagnoses, intraoperative tear characterisation, repair technique, rehabilitation exposure, and validated patient-reported and functional outcomes. Agent-specific analyses will be important given potential pharmacological differences between GLP-1RA. Finally, translational studies should move beyond acute repair models in young healthy rats toward chronic degenerative rotator cuff models, metabolically diseased animal models, and human rotator cuff tissue studies to clarify whether GLP-1RA-associated molecular effects translate into clinically meaningful changes in tendon degeneration, tendon–bone healing, or muscle recovery.

## Conclusion

This scoping review indicates that the evidence examining associations between GLP-1RA and rotator cuff disease remains limited. Available human observational evidence does not show an association between GLP-1RA use and increased short-term postoperative complications following rotator cuff repair. Preclinical rat repair models suggest that liraglutide may influence selected components of tendon–bone healing, muscle degeneration, and functional recovery under controlled experimental conditions. However, human observational studies suggest associations between GLP-1RA use and increased diagnosis of atraumatic rotator cuff tears and subsequent repair. Overall, the current evidence is insufficient to determine whether GLP-1RA exposure affects symptomatic rotator cuff disease progression, structural tendon healing, patient-reported outcomes, or functional recovery in routine clinical practice. Prospective studies with granular medication exposure data and validated clinical outcome measures are required to clarify these findings and inform evidence-based perioperative management.

## Supplementary Information

Supplementary Material 1.

Supplementary Material 2.

Supplementary Material 3.

Supplementary Material 4.

Supplementary Material 5.

## Data Availability

The datasets used and/or analysed during the current study are available from the corresponding author on reasonable request.

## Abbreviations

AMPK: AMP-activated protein kinase
BDNF: Brain-derived neurotrophic factor
BMI: Body mass index
CHOP: C/EBP homologous protein
CREB1: cAMP response element-binding protein 1
DM: Diabetes mellitus
DVT: Deep vein thrombosis
ED: Emergency department
ER: Endoplasmic reticulum
GIP: Glucose-dependent insulinotropic polypeptide
GLP-1: Glucagon-like peptide-1
GLP-1R: Glucagon-like peptide-1 receptor
GLP-1RA: Glucagon-like peptide-1 receptor agonist(s)
HbA1c: Glycated haemoglobin
MMP-13: Matrix metalloproteinase-13
PE: Pulmonary embolism
PKA: Protein kinase A
PRISMA-ScR: Preferred Reporting Items for Systematic Reviews and Meta-Analyses Extension for Scoping Reviews
qRT-PCR: Quantitative reverse transcription polymerase chain reaction
RCR: Rotator cuff repair
ROM: Range of motion
SD: Standard deviation
SGLT2i: Sodium-glucose cotransporter-2 inhibitor
T2DM: Type 2 diabetes mellitus
USA: United States of America
