# Supplementary material for: Glucagon-like peptide-1 receptor agonists and rotator cuff disease: a scoping review
Source: BMC Musculoskelet Disord. 2026 Jun 15;27:532. doi: 10.1186/s12891-026-10092-9 (PMC13295517; doi:10.1186/s12891-026-10092-9)
Supplement: Supplementary file 2 — Supplementary Material 2. [file 12891_2026_10092_MOESM2_ESM.docx]

**Supplementary Table 1. Search Strategies**

| **PubMed <inception to 2026 January 28>**   1. "glucagon-like peptide-1 receptor agonist"[tiab] OR "GLP-1"[tiab] OR "glucagon like peptide 1"[tiab] OR semaglutide[tiab] OR liraglutide[tiab] OR exenatide[tiab] OR dulaglutide[tiab] OR albiglutide[tiab] OR lixisenatide[tiab] OR tirzepatide[tiab] OR Ozempic[tiab] OR "Glucagon-Like Peptide-1 Receptor Agonists"[Mesh] (33492) 2. "rotator cuff"[tiab] OR "rotator cuff tear"[tiab] OR "rotator cuff disease"[tiab] OR supraspinatus[tiab] OR infraspinatus[tiab] OR subscapularis[tiab] OR teres minor[tiab] OR "shoulder tendon"[tiab] OR shoulder[tiab] OR "Rotator Cuff Injuries"[Mesh] (105835) 3. #1 AND #2 (16) |
| --- |
| **Embase <1974 to 2026 January 28>**   1. “glucagon like peptide 1”.mp or “GLP 1”.mp or “GLP-1”.mp or “glucagon-like peptide-1 receptor agonist”.mp or semaglutide.mp or liraglutide.mp or exenatide.mp or dulaglutide.mp or albiglutide.mp or lixisenatide.mp or tirzepatide.mp (77036) 2. exp glucagon like peptide 1 receptor agonist/ (73230) 3. 1 or 2 (79676) 4. “rotator cuff”.mp or “rotator cuff tear”.mp or “rotator cuff disease”.mp or supraspinatus.mp or infraspinatus.mp or subscapularis.mp or teres minor.mp or “shoulder”.mp (164037) 5. exp rotator cuff tear/ or exp shoulder/ or exp supraspinatus/ (109730) 6. 4 or 5 (196226) 7. 3 AND 6 (80) |
| **MEDLINE (via Ovid) <1946 to 2026 January 28>**   1. “glucagon like peptide 1”.mp or “GLP 1”.mp or “GLP-1”.mp or “glucagon-like peptide-1 receptor agonist”.mp or semaglutide.mp or liraglutide.mp or exenatide.mp or dulaglutide.mp or albiglutide.mp or lixisenatide.mp or tirzepatide.mp (35119) 2. exp glucagon like peptide 1 receptor agonist/ (9765) 3. 1 or 2 (35119) 4. “rotator cuff”.mp or “rotator cuff tear”.mp or “rotator cuff disease”.mp or supraspinatus.mp or infraspinatus.mp or subscapularis.mp or teres minor.mp or “shoulder”.mp (115629) 5. exp rotator cuff tear/ or exp shoulder/ or exp supraspinatus/ (28015) 6. 4 or 5 (115629) 7. 3 AND 6 (16) |
| **Cochrane <inception to 2026 January 28>**   1. (rotator cuff OR "rotator cuff tear" OR "rotator cuff disease"):ti,ab,kw (3439) 2. (supraspinatus OR infraspinatus OR subscapularis OR "teres minor"):ti,ab,kw (1164) 3. ("rotator cuff repair" OR "shoulder tendon" OR "shoulder surgery"):ti,ab,kw (2495) 4. #1 OR #2 OR #3 (5142) 5. ("glucagon-like peptide-1" OR GLP-1 OR "GLP 1"):ti,ab,kw (7327) 6. ((glucagon-like peptide-1 receptor NEXT agonist*) OR GLP-1RA*):ti,ab,kw (9085) 7. (semaglutide OR liraglutide OR exenatide OR dulaglutide OR albiglutide OR lixisenatide OR tirzepatide):ti,ab,kw (6720) 8. #5 OR #6 OR #7 (17279) 9. #4 AND #8 (6) |
