# Supplementary material for: Glucagon-like peptide-1 receptor agonists and rotator cuff disease: a scoping review
Source: BMC Musculoskelet Disord. 2026 Jun 15;27:532. doi: 10.1186/s12891-026-10092-9 (PMC13295517; doi:10.1186/s12891-026-10092-9)
Supplement: Supplementary file 3 — Supplementary Material 3. [file 12891_2026_10092_MOESM3_ESM.docx]

**Supplementary Table 2. Exposure definitions across included human studies and implications for outcome interpretation**

| Study | GLP-1RA exposure definition | Potential influence on outcome estimates |
| --- | --- | --- |
| Davis et al., 2026 | GLP-1RA exposure identified from database medication records. Agents included semaglutide, albiglutide, dulaglutide, liraglutide, lixisenatide, and exenatide. | Suitable for incident disease, but estimates may be affected by exposure misclassification, adherence uncertainty, treatment persistence, surveillance bias, and changes in weight, activity, or healthcare contact after GLP-1RA initiation. |
| Rasmussen & Ilyas, 2025 | Semaglutide, dulaglutide, or liraglutide for at least 3 months before rotator cuff repair. Tirzepatide was excluded because of dual GLP-1/GIP receptor activity. | The 3-month continuous-use definition increases exposure specificity but may select adherent or treatment-tolerant patients. It does not capture dose escalation, adherence, postoperative continuation, or perioperative interruption. |
| Seddio et al., 2025 | Semaglutide use documented within 1 year before arthroscopic rotator cuff repair. | The broad 1-year window may group active, remote, intermittent, or discontinued users. This may dilute associations with biologically meaningful perioperative exposure and limit inference about dose or duration. |
| Su et al., 2024 | New GLP-1RA use compared with new SGLT2 inhibitor use. First target-medication date was the index date; opposite drug-class use during baseline was excluded. | Active-comparator design reduces some indication confounding, but GLP-1RA and SGLT2i users may differ in renal function, cardiovascular risk, obesity severity, diabetes phenotype, prescribing indication, and healthcare utilisation. |
