# Supplementary material for: Glucagon-like peptide-1 receptor agonists and rotator cuff disease: a scoping review
Source: BMC Musculoskelet Disord. 2026 Jun 15;27:532. doi: 10.1186/s12891-026-10092-9 (PMC13295517; doi:10.1186/s12891-026-10092-9)
Supplement: Supplementary file 4 — Supplementary Material 4. [file 12891_2026_10092_MOESM4_ESM.docx]

**Supplementary Table 3. ROBINS-I assessment of human observational studies**

| Study | D1: Confounding | D2: Selection | D3: Classification | D4: Deviations | D5: Missing data | D6: Outcome measurement | D7: Reported result | Overall |
| --- | --- | --- | --- | --- | --- | --- | --- | --- |
| Davis et al., 2026 | Serious | Moderate | Moderate | Low | Low | Moderate | Moderate | Serious |
| Rasmussen and Ilyas, 2025 | Moderate | Moderate | Moderate | Low | Low | Moderate | Moderate | Moderate |
| Seddio et al., 2025 | Moderate | Moderate | Moderate | Low | Low | Moderate | Moderate | Moderate |
| Su et al., 2024 | Serious | Low | Moderate | Low | Low | Moderate | Moderate | Serious |

ROBINS-I domains: D1 = bias due to confounding; D2 = bias in selection of participants; D3 = bias in classification of interventions; D4 = bias due to deviations from intended interventions; D5 = bias due to missing data; D6 = bias in measurement of outcomes; D7 = bias in selection of the reported result.
