# Supplementary material for: Glucagon-like peptide-1 receptor agonists and rotator cuff disease: a scoping review
Source: BMC Musculoskelet Disord. 2026 Jun 15;27:532. doi: 10.1186/s12891-026-10092-9 (PMC13295517; doi:10.1186/s12891-026-10092-9)
Supplement: Supplementary file 5 — Supplementary Material 5. [file 12891_2026_10092_MOESM5_ESM.docx]

Supplementary Table 4. JBI critical appraisal of preclinical experimental studies

| Study | Q1: Cause/effect | Q2: Groups similar | Q3: Similar care | Q4: Control group | Q5: Multiple measures | Q6: Follow-up | Q7: Same measurement | Q8: Reliable measurement | Q9: Statistics | Overall appraisal |
| --- | --- | --- | --- | --- | --- | --- | --- | --- | --- | --- |
| Yoon et al., 2025 | Yes | Yes | Yes | Yes | Yes | Unclear | Yes | Yes | Yes | Moderate concern |
| Zhang et al., 2026 | Yes | Yes | Yes | Yes | No | Unclear | Yes | Yes | Yes | Moderate concern |

JBI quasi-experimental checklist items: Q1 = clear cause/effect relationship; Q2 = similarity of participants/groups; Q3 = similar treatment apart from exposure/intervention; Q4 = control group; Q5 = multiple measurements before and after exposure/intervention; Q6 = completeness of follow-up; Q7 = same outcome measurement across groups; Q8 = reliable outcome measurement; Q9 = appropriate statistical analysis.
